# Mesenchymal stem cells regulate the Th17/Treg cell balance partly through hepatocyte growth factor in vitro

**DOI:** 10.1186/s13287-020-01612-y

**Published:** 2020-02-28

**Authors:** Qi-Hong Chen, Fei Wu, Lei Liu, Han-bing Chen, Rui-Qiang Zheng, Hua-Ling Wang, Li-Na Yu

**Affiliations:** 1 Department of Critical Care Medicine, Jiangdu People’s Hospital of Yangzhou, Jiangdu People’s Hospital Affiliated with the Medical College of Yangzhou University, Yangzhou, Jiangsu People’s Republic of China; 2 Department of Intensive Care Unit, Affiliated Hospital of Yangzhou University, Yangzhou University, Yangzhou, 225001 Jiangsu People’s Republic of China; 3 Department of Critical Care Medicine, Northern Jiangsu People’s Hospital, Clinical Medical College of Yangzhou University, Yangzhou, Jiangsu People’s Republic of China; 4 Department of Cardiology, Northern Jiangsu People’s Hospital & Jiangdu People’s Hospital of Yangzhou, Yangzhou, Jiangsu People’s Republic of China

**Keywords:** Mesenchymal stem cells, Th17, Treg, Hepatocyte growth factor

## Abstract

**Introduction:**

Mesenchymal stem cells (MSCs) exert immunomodulatory functions by inducing the development and differentiation of naive T cells into T cells with an anti-inflammatory regulatory T cell (Treg) phenotype. Our previous study showed that hepatocyte growth factor (HGF) secreted by MSCs had immunomodulatory effects in the context of lipopolysaccharide (LPS) stimulation. We hypothesized that HGF is a key factor in the MSC-mediated regulation of the T helper 17 (Th17) cell/regulatory T (Treg) cell balance.

**Methods:**

We investigated the effects of MSCs on the differentiation of CD4+ T cells and the functions of Th17/Treg cells in response to LPS stimulation by performing in vitro coculture experiments. MSCs were added to the upper chambers of cell culture inserts, and CD4+ T cells were plated in the lower chambers, followed by treatment with LPS or an anti-HGF antibody. Th17 (CD4+CD3+RORrt+) and Treg (CD4+CD25+Foxp3+) cell frequencies were analysed by flow cytometry, and the expression of Th17 cell- and Treg cell-related cytokines in the CD4+ T cells or culture medium was measured by quantitative PCR (qPCR) and enzyme-linked immunosorbent assay (ELISA), respectively. Neutrophil functions were determined by flow cytometry after a coculture with Th17/Treg cells.

**Results:**

The percentage of CD4+CD25+Foxp3+ cells was significantly increased in the CD4+ T cell population, while the percentage of CD4+CD3+RORrt+ cells was significantly decreased after MSC coculture. However, the MSC-induced effect was significantly inhibited by the anti-HGF antibody (*p* < 0.05). Furthermore, MSCs significantly inhibited the CD4+ T cell expression of IL-17 and IL-6 but increased the expression of IL-10 (*p* < 0.05 or *p* < 0.01); these effects were inhibited by the anti-HGF antibody (*p* < 0.05). In addition, CD4+ T cells cocultured with MSCs significantly inhibited neutrophil phagocytic and oxidative burst activities (*p* < 0.05 or *p* < 0.01); however, these MSC-induced effects were inhibited by the anti-HGF antibody (*p* < 0.05).

**Conclusion:**

These data suggested that MSCs induced the conversion of fully differentiated Th17 cells into functional Treg cells and thereby modulated the Th17/Treg cell balance in the CD4+ T cell population, which was partly attributed to HGF secreted by the MSCs.

## Introduction

Acute respiratory distress syndrome (ARDS) is characterized by increased lung permeability, pulmonary oedema and diffuse inflammation, which lead to disruption of alveolar capillary membranes [1]. Many studies have indicated that excessive activation of multiple inflammatory cell types and release of inflammatory mediators play vital roles in the development of ARDS [2, 3]. Among these cell types, CD4+ T cells play an important role in the pathogenesis of ARDS [4].

Regulatory T (Treg) cells have anti-inflammatory roles mainly mediated by contact-dependent suppression and the release of cytokines that impact other immune cells, including CD4+ T cells [5]. Studies have shown that the alveolar recruitment of Treg cells contributes to the resolution of lung inflammation in mice or patients with ARDS [6, 7]. In addition to Treg cells, Th17 cells, another subset of CD4+ T cells, play a potent proinflammatory role in the immune system through production of the signature cytokine IL-17 and other inflammatory mediators, including IL-6 and TNF-α [8]. Yu et al. showed that the ratio of Th17/Treg cells was closely related to illness severity and 28-day mortality in ARDS patients [9].

Mesenchymal stem cells (MSCs) have extensive immunomodulatory effects, such as inhibiting T cell proliferation, activation and cytokine production [10, 11]. Our previous study showed that MSCs exerted immunomodulatory effects through paracrine hepatocyte growth factor (HGF) signalling under LPS stimulation [12]. A study showed that Tregs played an important role in MSC-mediated immunomodulation under inflammatory conditions [13]. In addition, MSCs mediate the adhesion of Th17 cells to exert anti-inflammatory effects through the induction of a regulatory T cell phenotype in these cells [14]. However, the detailed mechanism by which MSCs regulate T cells remains poorly defined. Therefore, we hypothesized that HGF is a key factor in the MSC-mediated regulation of the Th17/Treg cell balance. To verify this hypothesis, we investigated the effects of MSCs on the differentiation of CD4+ T cells and the functions of fully differentiated Th17 cells and Treg cells stimulated with LPS by performing in vitro coculture experiments.

## Materials and methods

### MSC culture

**Fig. 1:**
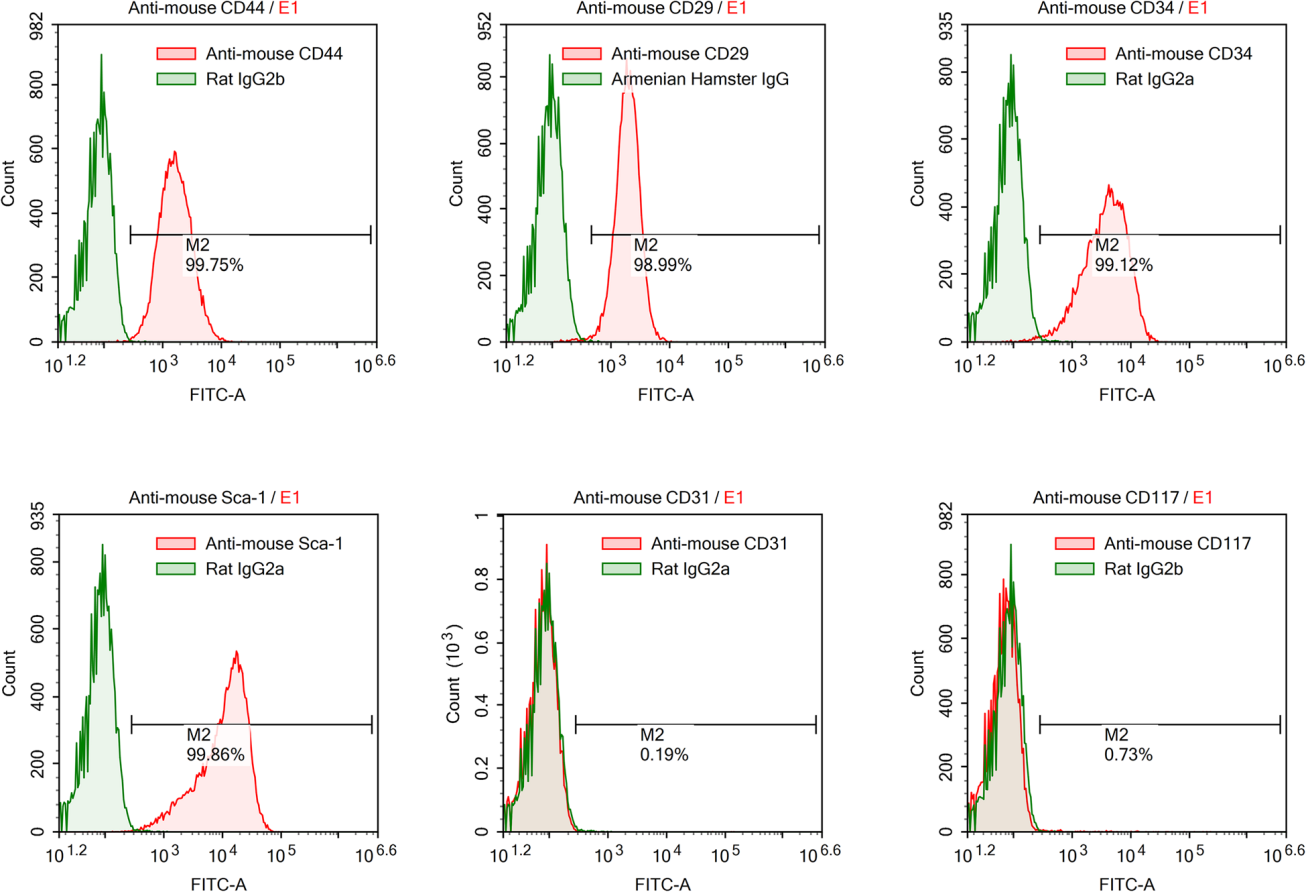
Flow cytometry identification of mouse mesenchymal stem cells (MSCs). Cell surface markers of MSCs, including CD44, CD29, CD117, CD31, CD34 and Sca-1, were analysed with flow cytometry. Red lines represent the isotype controls. Cytofluorometric analyses showed the presence of several molecules such as CD44, CD29, CD34 and Sca-1 but not the presence of CD117 or CD31

C57BL/6 mice (6–8 weeks old) were used to obtain bone marrow-derived MSCs. MSCs were purchased from Cyagen Biosciences Inc. (Guangzhou, China). The surface markers of MSCs (CD29, CD44, CD117, CD31, CD34 and Sca-1) were detected by flow cytometry to confirm stem cell identity (Abcam, Hong Kong). Cytofluorometric analyses showed the presence of several molecules such as CD44, CD29, CD34 and Sca-1 but not the presence of CD117 or CD31 (Fig. 1). MSCs were cultured in MSC growth medium (Cyagen Biosciences Inc.). All cells were cultured in a humidified 5% CO_2_ incubator at 37 °C. The culture medium was changed every 3 days, and the cells were used at passage 3–7 for all experiments. MSCs were cultured at a density of 1 × 10^3^, 5 × 10^3^, 1 × 10^4^, 5 × 10^4^ or 1 × 10^5^ cells per well. After culturing for 24 h, MSCs were treated with 100 ng/ml LPS (Sigma, USA).

### CD4+ T cell culture

CD4+ T cells were isolated from mouse spleens and sorted using magnetic beads (Miltenyi Biotec, Bergisch Gladbach, Germany). Briefly, the spleens were obtained from C57BL/6 mice euthanized by cervical dislocation. Then, the mouse spleens were mechanically dissociated, and dispersed single cells were isolated in a lymphocyte separation solution after filtration through nylon mesh. The single-cell suspension was resuspended in PBS after centrifugation. CD4+ T cells were then negatively selected with a magnetic activated cell sorting (MACS) system (eBioscience), evaluated by flow cytometry, and examined under a light microscope following staining with 2 g/L Trypan blue. All cells were cultured in a humidified 5% CO_2_ incubator at 37 °C. The culture medium was changed every 3 days, and the cells were used at passage 3–7 for all experiments.

### MSC and CD4+ T cell cocultures

MSCs were added to the upper chambers of 0.4-μm cell culture inserts (membrane diameter, 24 mm; catalogue number, 3412; Corning, USA) at a density of 50,000 cells per insert. After the MSCs were grown to 50% confluence, CD4+ T cells were plated in the lower chambers at a density of 50,000 cells/cm^2^. Then, the medium was changed to DMEM/F12 supplemented with 10% FBS (Wisent Inc., China). After the CD4+ T cells were cultured for approximately 10 days, LPS (100 ng/ml, Sigma) or a polyclonal rabbit anti-HGF antibody (ab83760, 100 ng/ml) was added to the culture system. After 24 h of coculture with LPS or the anti-HGF antibody, the CD4+ T cells or conditioned medium supernatants were collected to evaluate CD4+ T cell cytokine expression and T cell subset-specific markers. All experiments were performed in triplicate.

### Th17/Treg cell determination

CD4+ T cells from C57BL/6 mice were cocultured with MSCs. LPS (100 ng/ml) and 100 pg/ml recombinant mouse HGF (rmHGF; Sino Biological, China) or the polyclonal rabbit anti-HGF antibody (ab83760, 100 ng/ml) were added to the cocultures of stimulated CD4+ T cells. The proportions of CD4+ T cells expressing RORγt or Foxp3 and CD25 (eBioscience) were determined after a 96-h incubation period by flow cytometry according to the manufacturer’s instructions. Th17 cells were defined as CD4+CD3^+^RORγt^+^ cells, and Treg cells were defined as CD4^+^CD25^+^Foxp3^+^ cells.

### Detection of gene expression

After 10 days of coculture with MSCs, CD4+ T cells were collected to evaluate the expression of Th17 cell- and Treg cell-related cytokines by quantitative PCR (qPCR). Total RNA was extracted from stimulated CD4+ T cells using Extract RNA reagent (Thermo) according to the instructions of the manufacturer. The isolated total RNA was reverse transcribed with an MMLV RT kit (Fermentas). All primers sequences can be seen in Additional file 1. Quantitative PCR (qPCR) was performed with 2 mL cDNA and SYBR Green Master Mix (Thermo) on a Light Cycler system using forward and reverse primers specific for target genes. The reaction cycles were performed under the following conditions: 40 cycles of 95 °C for 15 s and 60 °C for 45 s, followed by 95 °C for 15 s and 60 °C for 1 min, and then 95 °C for 15 s and 60 °C for 15 s. Changes in target gene expression levels were calculated as fold differences using ABI Prism 7300 SDS software. All primer sequences are available upon request.

### Measurement of supernatant cytokine levels

Culture supernatants were collected and stored at − 80 °C after treatment. Enzyme-linked immunosorbent assay (ELISA) analyses were performed according to the instructions of the ELISA kit manufacturers. Quantitative analysis of HGF, IL-17, IL-6, TGF-β and IL-10 (YH Biosearch Laboratory, Shanghai, China) levels in the collected conditioned supernatants was conducted to evaluate cytokine changes in CD4+ T cell subsets. The OD value at 450 nm was measured. The concentrations of IL-17, IL-6, TGF-β and IL-10 were calculated according to the appropriate standard curve.

### Phagocytic and oxidative burst activities

CD4+ T cells cocultured with MSCs were collected and cocultured with neutrophils at a ratio of 10:1, and then the OVA 323-339 peptide (200 nM) was added for 7 days. The neutrophils were isolated using a neutrophil isolation kit (Weigelas, China) and seeded in 96-well plates at 1 × 10^5^ cells/200 μl. After an incubation, dextran-FITC (100 μg/ml) or 20 mg/ml PMA (500 nmol/l) was added to the neutrophil suspensions to measure phagocytic activity or oxidative burst activity, respectively. Then, the treated suspensions were incubated at 37 °C for 4 h and centrifuged three times in cold PBS; fluorescence intensity was measured by flow cytometry.

### Monocyte activation detection

CD4+ T cells cocultured with MSCs were collected and cocultured with monocytes at a ratio of 10:1, and then the OVA 323-339 peptide (200 nM) was added for 7 days. The monocytes were isolated using a monocyte isolation kit (eBioscience) and seeded in 96-well plates at 1 × 10^5^ cells/200 μl. The proportions of monocytes expressing CD40, CD80, TLR2 or MHC-II were determined after a 96-h incubation period by flow cytometry according to the manufacturer’s instructions.

### Statistical analysis

All results are expressed as the mean ± standard deviation (SD). Statistical analyses were performed using the SPSS 16.0 software package. For group comparisons, one-way analysis of variance (ANOVA) followed by Tukey’s test was used. A value of *p* < 0.05 was considered statistically significant.

## Results

### Th17/Treg cell differentiation of CD4+ T cells

**Fig. 2:**
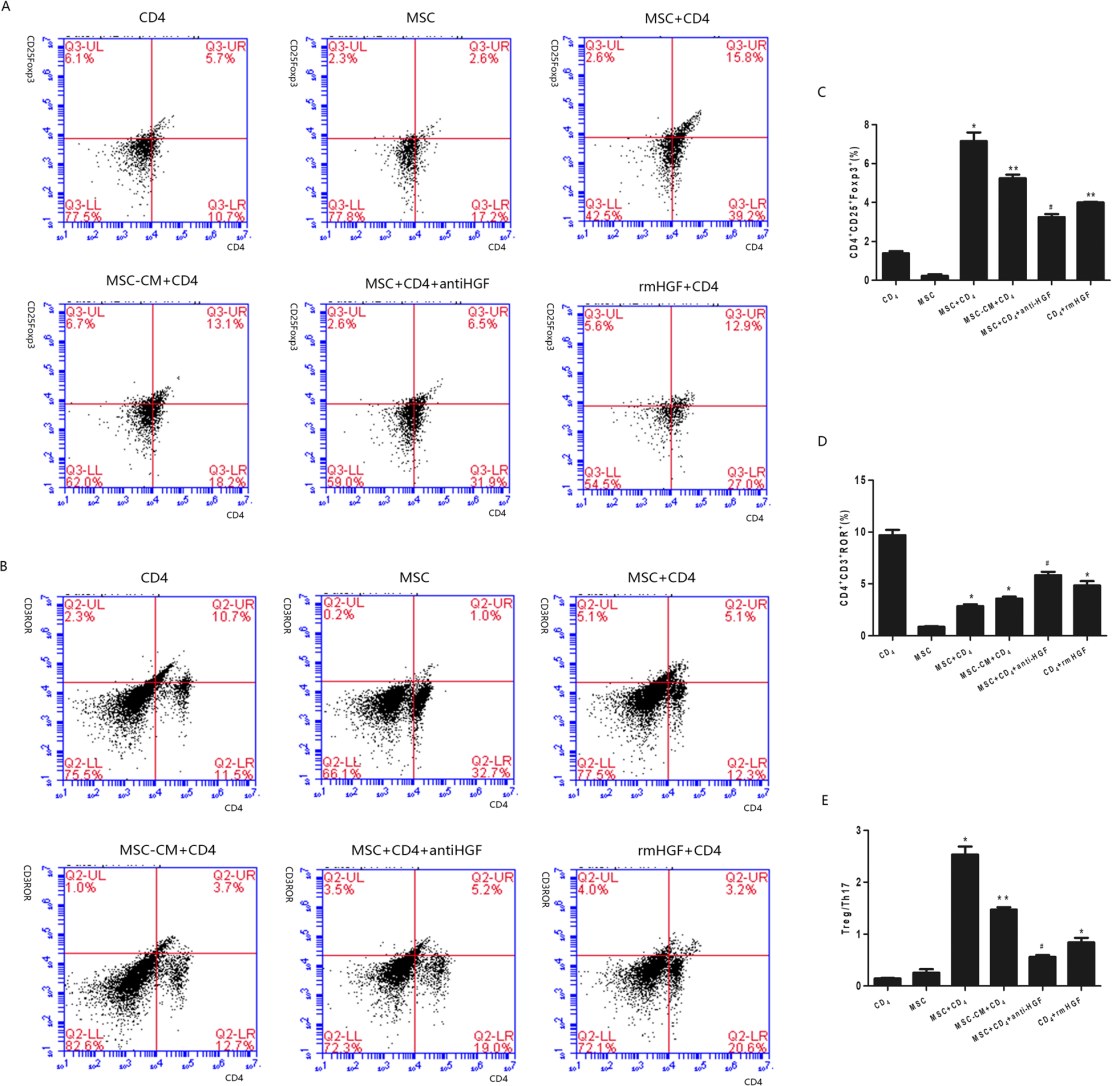
The modulatory effect of HGF secreted by MSCs on Th17/Treg cell differentiation in CD4+ T cells analysed by flow cytometry. The results showed that the percentage of CD4+CD25+Foxp3+ cells was significantly increased in the CD4+ T cell population, but the MSC-induced effect was significantly inhibited by an anti-HGF antibody (*p* < 0.05, **a**, **c**). The percentage of CD4+CD3+RORrt+ cells was significantly decreased after MSC coculture. However, the MSC-induced effect was significantly inhibited by the anti-HGF antibody (*p* < 0.05, **b**, **d**) (*n* = 3; *p < 0.05 or **p < 0.01 vs. the CD4+ group; ^#^*p* < 0.05 vs. the MSC+CD4+ group). MSCs, mesenchymal stem cells; rmHGF, recombinant mouse hepatocyte growth factor

To clarify that MSCs regulate the expression of the transcription factor Foxp3 in CD25+ T cells and that of RORγt in CD3+ T cells, which control the development of Treg cells and Th17 cells, respectively, CD4+ T cells were cocultured with MSCs, and the percentages of CD4+CD25+Foxp3+ and CD4+CD3+RORγt+ cells were determined by flow cytometry. The results showed that the percentage of CD4+CD25+Foxp3+ cells was significantly increased in the CD4+ T cell population, while the percentage of CD4+CD3+RORrt+ cells was significantly decreased after MSC coculture. However, the MSC-induced effect was significantly inhibited by an anti-HGF antibody (*p* < 0.05, Fig. 2). These results indicated that MSCs regulated Th17/Treg cell differentiation partly via HGF.

### Cytokine mRNA expression in CD4+ T cells

**Fig. 3:**
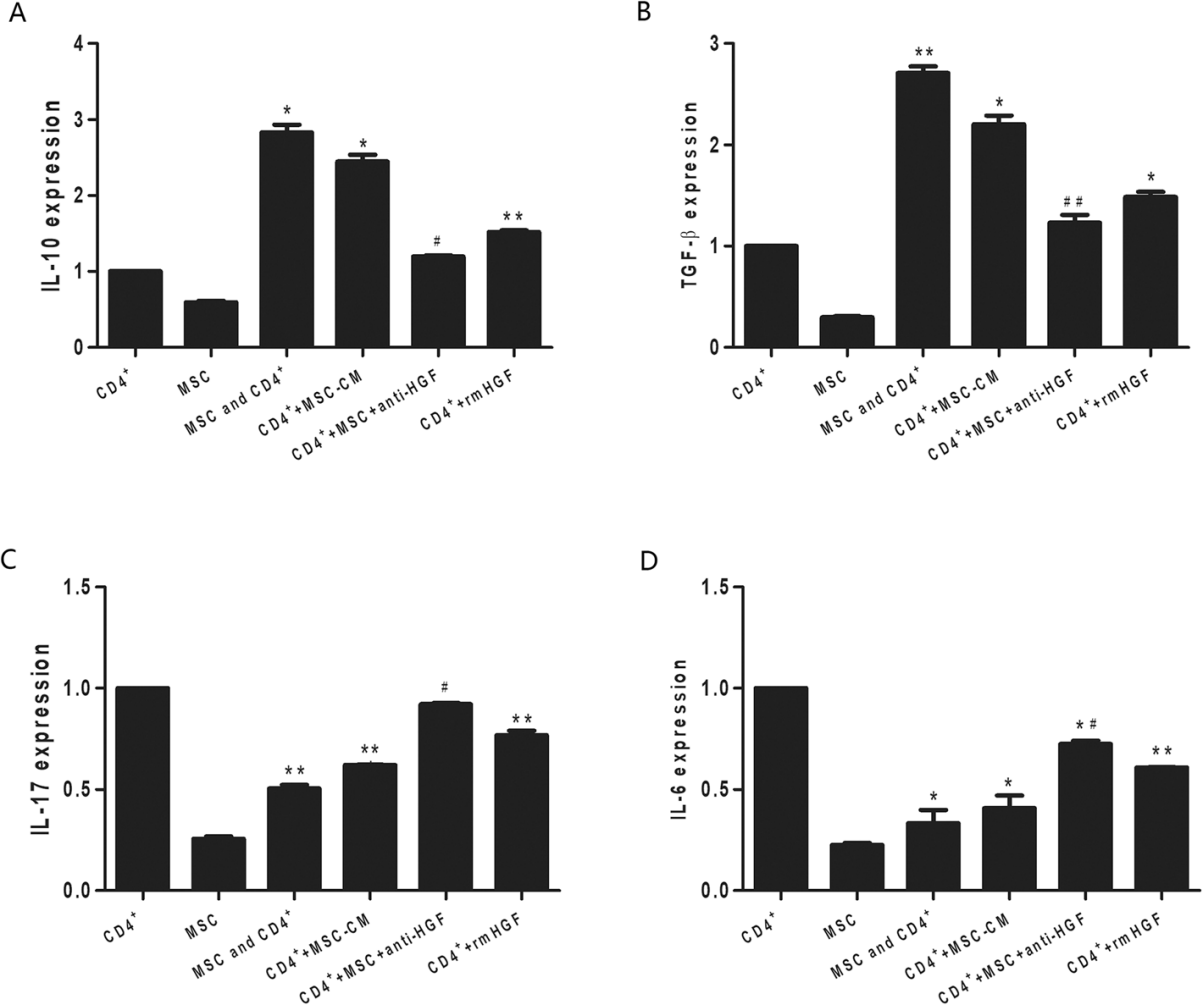
The regulatory effect of HGF secreted by MSCs on cytokine mRNA expression in CD4+ T cells analysed by qPCR. The results showed that MSCs significantly increased IL-10 (*p* < 0.05, **a**) and TGF-β (*p* < 0.01, **b**) mRNA expression and inhibited IL-17 (*p* < 0.01, **c**) and IL-6 (*p* < 0.05, **d**) mRNA expression in CD4+ T cells. However, the MSC-induced effects were inhibited by an anti-HGF antibody (*p* < 0.05 or *p* < 0.01) (*n* = 3; *p < 0.05 or **p < 0.01 vs. the CD4+ group; ^#^*p* < 0.05 or ^##^*p* < 0.01 vs. the MSC+CD4+ group). MSCs, mesenchymal stem cells; rmHGF, recombinant mouse hepatocyte growth factor; qPCR, quantitative polymerase chain reaction

We tested the effects of MSCs on the mRNA expression of proinflammatory cytokines and anti-inflammatory factors in CD4+ T cells by qPCR. The results showed that MSCs significantly inhibited IL-17 and IL-6 mRNA expression and increased IL-10 and TGF-β mRNA expression in CD4+ T cells. However, the MSC-induced effect was inhibited by the anti-HGF antibody (*p* < 0.05 or *p* < 0.01, Fig. 3). These results suggested that MSCs inhibited the CD4+ T cell expression of proinflammatory cytokines and increased the CD4+ T cell expression of anti-inflammatory factors partly through HGF.

### Cytokine production in MSCs or CD4+ T cells

HGF levels were detected in MSC culture supernatants by ELISA. The results showed that MSCs secreted HGF after LPS stimulation in an MSC number-dependent manner (Additional file 2). To demonstrate the effects of MSCs on the cytokine production of CD4+ T cells, purified naive CD4+ splenocytes were activated with allogeneic cells in the presence of MSCs and LPS. As demonstrated in Fig. 3, the MSCs significantly inhibited the CD4+ T cell secretion of IL-17 and IL-6 and increased the CD4+ T cell secretion of IL-10 and TGF-β. However, the MSC-induced effect was inhibited by the anti-HGF antibody (*p* < 0.05 or *p* < 0.01, Fig. 3). These results indicated that MSCs inhibited proinflammatory cytokine secretion by CD4+ T cells and increased anti-inflammatory factor secretion by CD4+ T cells partly through HGF.

### Neutrophil activities modulated by CD4+ T cells

**Fig. 4:**
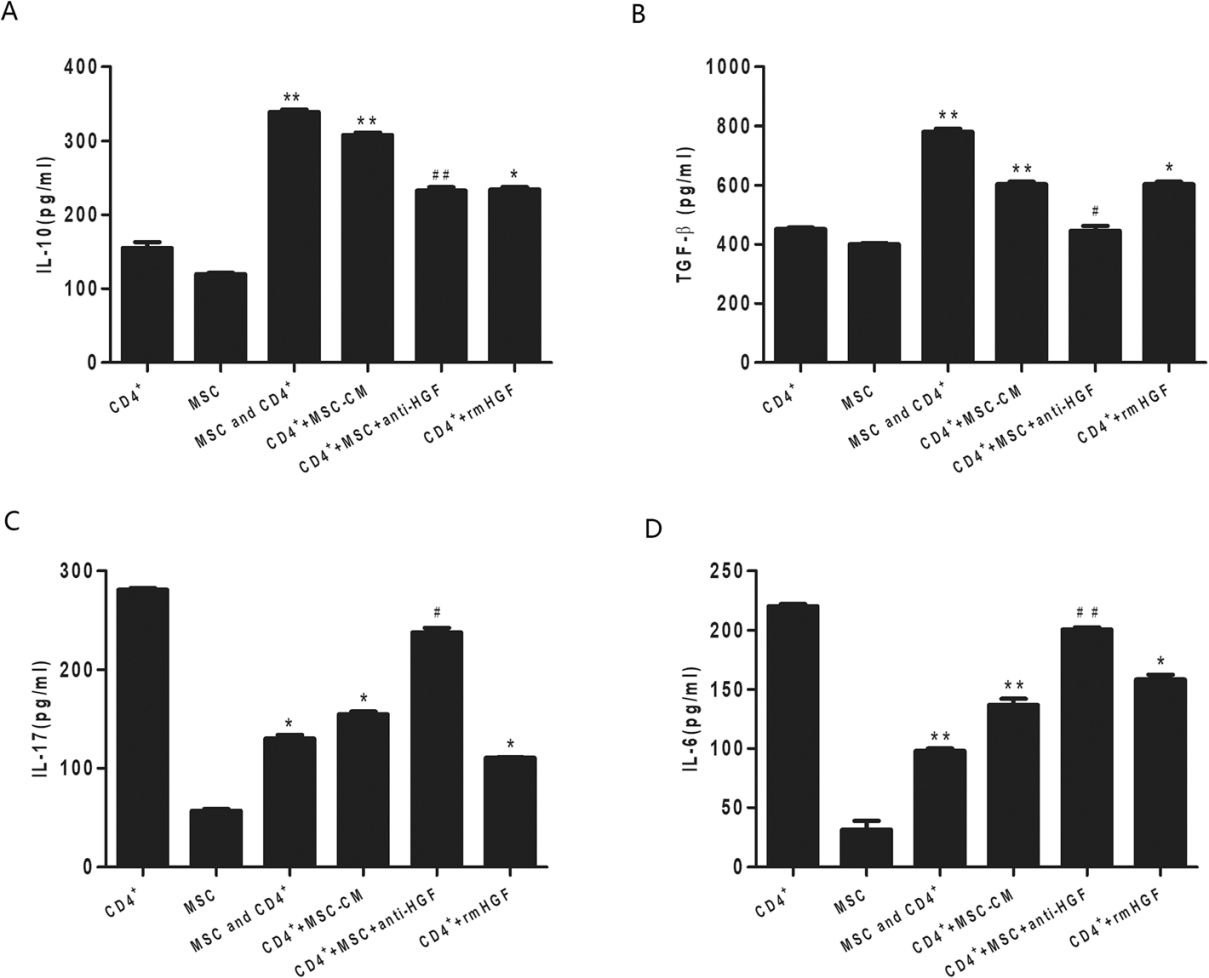
The regulatory effect of HGF secreted by MSCs on cytokine production in CD4+ T cells detected by ELISA. We found that MSCs significantly increased the CD4+ T cell secretion of IL-10 (*p* < 0.01, **a**) and TGF-β (*p* < 0.01, **b**) but inhibited the CD4+ T cell secretion of IL-17 (*p* < 0.05, **c**) and IL-6 (*p* < 0.01, **d**). However, the MSC-induced effects were inhibited by an anti-HGF antibody (*p* < 0.05 or *p* < 0.01) (*n* = 3; *p < 0.05 or **p < 0.01 vs. the CD4+ group; ^#^*p* < 0.05 or ^##^*p* < 0.01 vs. the MSC+CD4+ group). MSCs, mesenchymal stem cells; rmHGF, recombinant mouse hepatocyte growth factor

There are many studies on the roles of neutrophil functions, such as oxidative burst and phagocytosis, in critically ill patients. In this study, disturbances in the oxidative burst and phagocytic activities of neutrophils were detected by flow cytometry according to manufacturer specifications. The results showed that CD4+ T cells cocultured with MSCs significantly inhibited neutrophil phagocytic and oxidative burst activities (*p* < 0.05 or *p* < 0.01, Fig. 4). However, the effect mediated by CD4+ T cells was inhibited by the anti-HGF antibody (*p* < 0.05). These results indicated that MSCs induced CD4+ T cells to inhibit neutrophil phagocytic and oxidative burst activities partly via HGF.

### Monocyte activation by CD4+ T cells

**Fig. 5:**
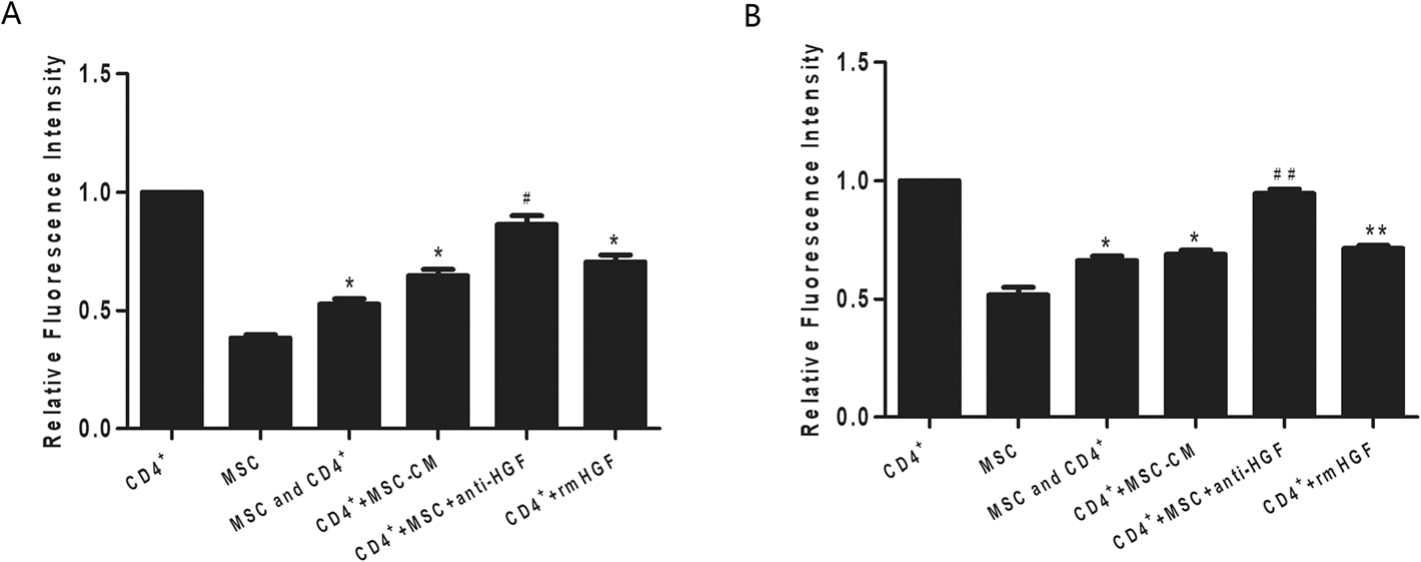
The regulatory effect of HGF secreted by MSCs on neutrophil activities in cells cocultured with CD4+ T cells and analysed with flow cytometry. The results indicated that CD4+ T cells cocultured with MSCs significantly inhibited neutrophil phagocytic (*p* < 0.05, **a**) and oxidative burst activities (*p* < 0.01, **b**). However, the effects of the CD4+ T cells were inhibited by an anti-HGF antibody (*p* < 0.05 or *p* < 0.01) (*n* = 3; *p < 0.05 or **p < 0.01 vs. the CD4+ group; ^#^*p* < 0.05 or ^##^*p* < 0.01 vs. the MSC+CD4+ group). MSCs, mesenchymal stem cells; rmHGF, recombinant mouse hepatocyte growth factor

**Fig. 6:**
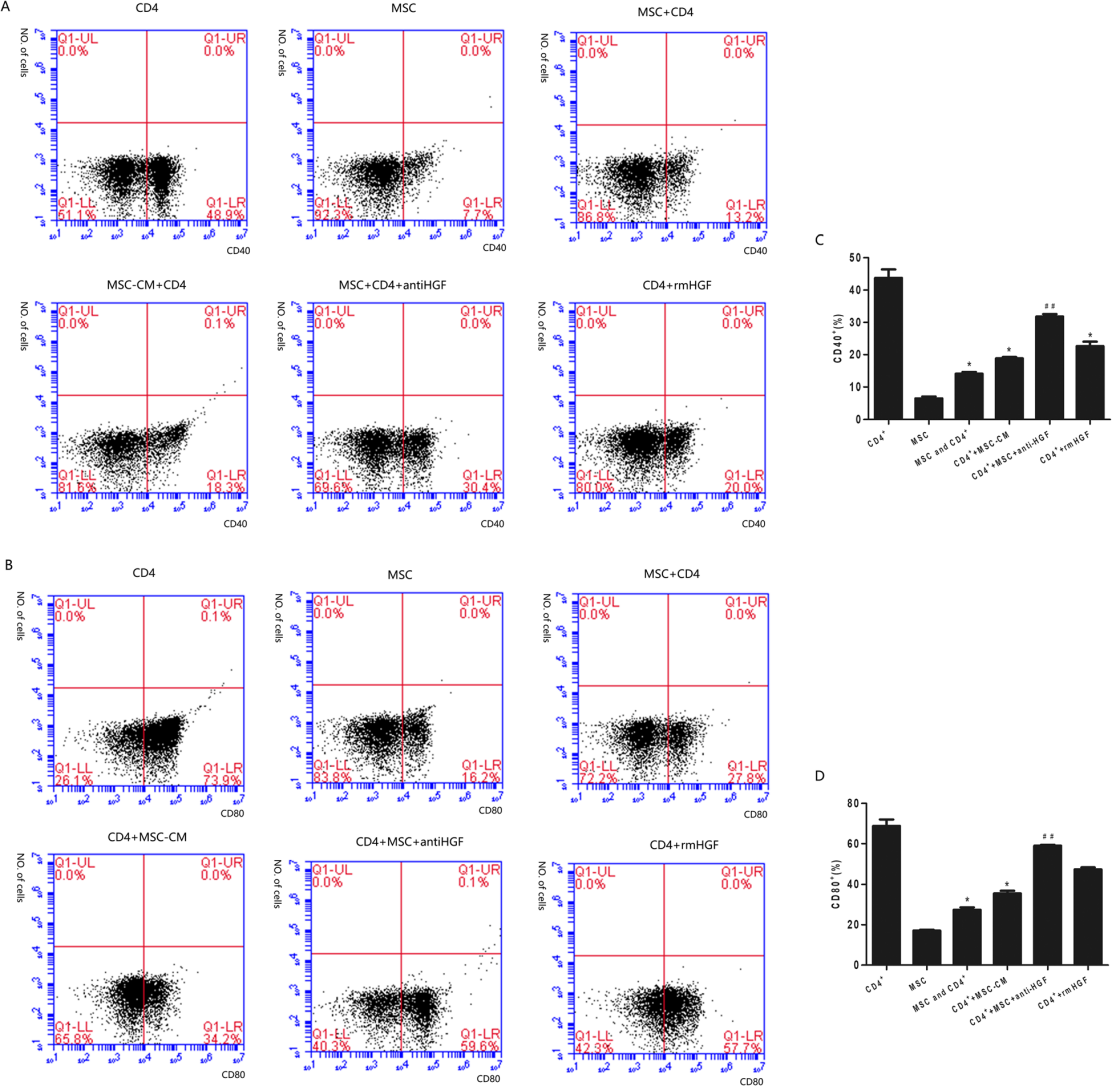
The regulatory effect of HGF secreted by MSCs on CD40+ and CD80+ monocyte activation in cocultures with CD4+ T cells and analysed with flow cytometry. The results showed that after being cocultured with MSCs, CD4+ T cells significantly inhibited monocyte expression of CD40 (*p* < 0.05, **a**) and CD80 (*p* < 0.05, **b**). However, the CD4+ T cell-mediated effect was inhibited by an anti-HGF antibody (*p* < 0.01) (*n* = 3; *p < 0.05 vs. the CD4+ group; ^##^*p* < 0.01 vs. the MSC+CD4+ group). MSCs, mesenchymal stem cells; rmHGF, recombinant mouse hepatocyte growth factor

**Fig. 7:**
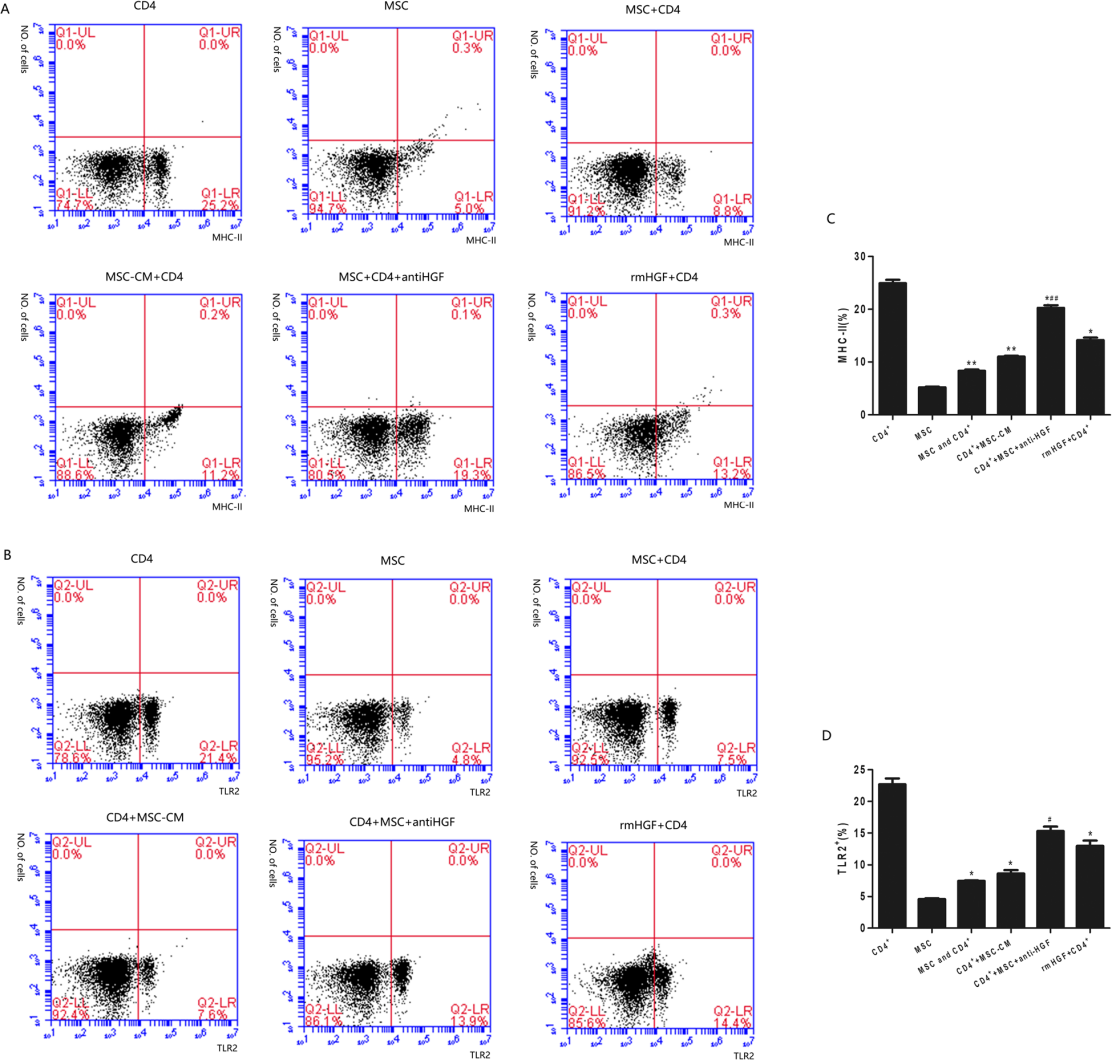
The regulatory effect of HGF secreted by MSCs on MHC-II and TLR-2 expression in monocytes cocultured with CD4+ T cells and analysed with flow cytometry. The results showed that after being cocultured with MSCs, CD4+ T cells significantly inhibited monocyte expression of MHC-II (*p* < 0.01, **a**) and TLR2 (*p* < 0.05, **b**). However, the CD4+ T cell-mediated effect was inhibited by an anti-HGF antibody (*p* < 0.05 or *p* < 0.01) (*n* = 3; *p < 0.05 or **p < 0.01 vs. the CD4+ group; ^##^*p* < 0.01 vs. the MSC+CD4+ group). MSCs, mesenchymal stem cells; rmHGF, recombinant mouse hepatocyte growth factor

Monocytes are among the first responders to arrive in the lungs following acute lung injury, and they are known to assume an activated phenotype defined by the expression of CD40, CD80, TLR2 and MHC-II, which stimulate adaptive immune responses. In this study, monocyte expression of CD40, CD80, TLR2 and MHC-II was analysed using flow cytometry following coculture of the monocytes with stimulated CD4+ T cells. The results showed that after being cocultured with MSCs, CD4+ T cells significantly inhibited monocyte expression of CD40, CD80, TLR2 and MHC-II. However, the CD4+ T cell-mediated effect was inhibited by the anti-HGF antibody (*p* < 0.05 or *p* < 0.01, Figs. 5 and 6). These results suggested that MSCs induced CD4+ T cells to inhibit monocyte activation partly via HGF (Fig. 7).

## Discussion

Mesenchymal stem cells (MSCs) have extensive immunomodulatory effects, such as inhibiting T cell proliferation, activation and cytokine production; however, the detailed mechanism by which MSCs regulate T cells remains poorly defined [15, 16]. In the present study, we found that the percentage of CD4+CD25+Foxp3+ cells in the CD4+ T cell population was significantly decreased, while the percentage of CD4+CD3+RORrt+ cells was significantly increased after a coculture with MSCs. Furthermore, MSCs significantly inhibited the CD4+ T cell expression of IL-17 and IL-6 but increased the expression of IL-10. In addition, CD4+ T cells cocultured with MSCs significantly inhibited neutrophil phagocytic and oxidative burst activities. However, the effects of MSCs described above were inhibited by an anti-HGF antibody.

Many studies have suggested that the activation of multiple inflammatory cell types and release of inflammatory mediators play vital roles in the development of ARDS [17, 18]. Among those cell types, CD4+ T cells play a key role in the pathogenesis of ARDS [8]. Th17 and Treg cell numbers are increased in the peripheral blood of patients with early-stage ARDS, and an increased Th17/Treg cell ratio is associated with a relatively poor prognosis [9]. Thus, strategies designed to restore the Th17/Treg cell balance may be an effective therapeutic approach for ARDS.

MSCs have extensive immunomodulatory effects, such as inhibiting T cell proliferation, activation and cytokine production [19]. A study showed that cell-to-cell contact, inhibitory molecule production and regulatory T cell (Treg) induction are implicated in the immunomodulatory mechanism mediated by MSCs [20]. Under inflammatory conditions, MSCs exert anti-inflammatory effects through the induction of a regulatory T cell phenotype in CD4+ T cells [21]. We investigated the effects of MSCs on the differentiation of CD4+ T cells and the functions of fully differentiated Th17 cells and Treg cells treated with LPS stimulation by performing in vitro coculture experiments. The results showed that MSCs regulated the polarization of naive T cells into regulatory T cells and reduced the Th17/Treg cell ratio. Therefore, MSCs induce the conversion of fully differentiated Th17 cells into functional Treg cells, thereby regulating Th17/Treg cell differentiation in CD4+ T cells stimulated with LPS.

Neutrophils are professional phagocytes that play a vital role in host defence against infection and are important in the pathogenesis of many inflammatory diseases, such as ARDS [22]. The transmigration of neutrophils through the endothelium promotes the acute phase of pulmonary inflammation [23]. Two critical neutrophil effector functions are the generation of an oxidative burst and phagocytosis, and neutrophils react with excessive proinflammatory cytokine release and oxidative bursts, which in turn further aggravate the overall cellular inflammatory response and lung tissue injury [24–27]. This study indicated that MSCs induced CD4+ T cells to inhibit neutrophil phagocytic and oxidative burst activities, which suggested that the immunomodulatory effects of MSCs were involved in the regulation of neutrophil activation and function. These findings provide a new way for us to explore the mechanism underlying MSC-mediated immune regulation.

HGF is a multifunctional, mesenchyme-derived pleiotropic factor secreted by several cell types [25, 28] that regulates many biological events, such as cell mitogenesis, organogenesis, morphogenesis, cell survival and vascular endothelial barrier function [29, 30]. However, the short half-life of the HGF protein in vivo requires the administration of multiple doses, and intravenous drug delivery cannot guarantee the trafficking of HGF to injured tissues. Recently, the therapeutic effect of HGF secreted by MSCs on ARDS was observed, and related mechanisms were investigated. Our previous study showed that hepatocyte growth factor secreted by MSCs exerted immunomodulatory effects through anti-inflammatory mediator activation [31, 32]. This study showed that MSCs regulated the Th17/Treg cell balance of CD4+ T cells stimulated with LPS. However, the immunomodulatory effects of MSCs were obviously inhibited by an anti-HGF antibody, which suggested that HGF secreted by MSCs might be a key factor in MSC-mediated immunoregulation. This study provides an important theoretical basis for the future use of MSCs in the treatment of ARDS patients.

There is a limitation to this study. This study suggested that MSCs regulated the Th17/Treg cell balance of CD4+ T cells stimulated with LPS, which was mainly attributed to HGF secreted by the MSCs. However, the study was performed in vitro. Our future study will perform further research in vivo to verify the role of HGF in the immunomodulatory effects of MSCs.

## Conclusion

These data suggested that MSCs induced the conversion of fully differentiated Th17 cells into functional Treg cells and thereby modulated the Th17/Treg balance in a CD4+ T cell population stimulated with LPS. Furthermore, the immunomodulatory effects of MSCs involved the regulation of neutrophil activation and functions. These immunomodulatory effects of MSCs were obviously inhibited by an anti-HGF antibody, which suggested that MSCs regulated the Th17/Treg cell balance partly through HGF. This study provides a potential therapeutic target for acute lung injury treatment.

## Supplementary information

**Additional file 1.** Primers sequences for qPCR detection of IL-17, IL-6, IL-10 and TGF-β. qPCR, quantitative polymerase chain reaction.

**Additional file 2.** Effects of different numbers of MSCs on secreted HGF levels measured by ELISA. MSCs, mesenchymal stem cells; rmHGF, recombinant mouse hepatocyte growth factor; ELISA, enzyme-linked immunosorbent assay.

## Data Availability

Please contact the authors for data requests.

## Abbreviations

ALI: Acute lung injury
ARDS: Acute respiratory distress syndrome
ELISA: Enzyme-linked immunosorbent assay
HGF: Hepatocyte growth factor
LPS: Lipopolysaccharide
MSCs: Mesenchymal stem cells
rmHGF: Recombinant mouse HGF
Th17: T helper 17
Treg: Regulatory T cell
