# Supplementary material for: Mesenchymal stem cells regulate the Th17/Treg cell balance partly through hepatocyte growth factor in vitro
Source: Stem Cell Res Ther. 2020 Feb 28;11:91. doi: 10.1186/s13287-020-01612-y (PMC7049226; doi:10.1186/s13287-020-01612-y)
Supplement: Supplementary file 1 — Additional file 1. Primers sequences for qPCR detection of IL-17, IL-6, IL-10 and TGF-β. qPCR, quantitative polymerase chain reaction. [file 13287_2020_1612_MOESM1_ESM.doc]

**Mus musculus interleukin 17A (Il17a), mRNA**

NM_010552.3

Primer F 5' GCCCTCAGACTACCTCAACC 3'

Primer R 5' CACCCACCAGCATCTTCTC 3'

Pos: 252---490 C

Amplified product: Size: 239 bps Product GC 56%

**Mus musculus interleukin 10 (Il10), mRNA**

NM_010548.2

Primer F 5' CTTTGCTATGGTGTCCTTTC 3'

Primer R 5' ATCTCCCTGGTTTCTCTTC 3'

Pos: 983-----1084 C

# Amplified product: Size: 102 bps Product GC 46%

**Mus musculus transforming growth factor, beta 1 (Tgfb1), mRNA**

NM_011577.1

Primer F 5' AAGGACCTGGGTTGGAAGTG 3'

Primer R 5' TGGTTGTAGAGGGCAAGGAC 3'

Pos: 1777-1901

Amplified product: Size: 125 bps Product GC 56%

**Mus musculus interleukin 6 (Il6), mRNA**

**NM_031168.1**

Primer F 5' CAAAGCCAGAGTCCTTCAG 3'

Primer R 5' GATGGTCTTGGTCCTTAGC 3'

Pos: 442-601

Amplified product: Size: 160 bps Product GC 41%

**Mus musculus glyceraldehyde-3-phosphate dehydrogenase (Gapdh), mRNA**

NM_008084.2

Primer F 5' ATCACTGCCACCCAGAAG 3'

Primer R 5' TCCACGACGGACACATTG 3'

Pos: 585-775

Amplified product: Size: 191 bps Product GC 60%
