# Supplementary figures and images for: Mesenchymal stem cells regulate the Th17/Treg cell balance partly through hepatocyte growth factor in vitro
Source: Stem Cell Res Ther. 2020 Feb 28;11:91. doi: 10.1186/s13287-020-01612-y (PMC7049226; doi:10.1186/s13287-020-01612-y)

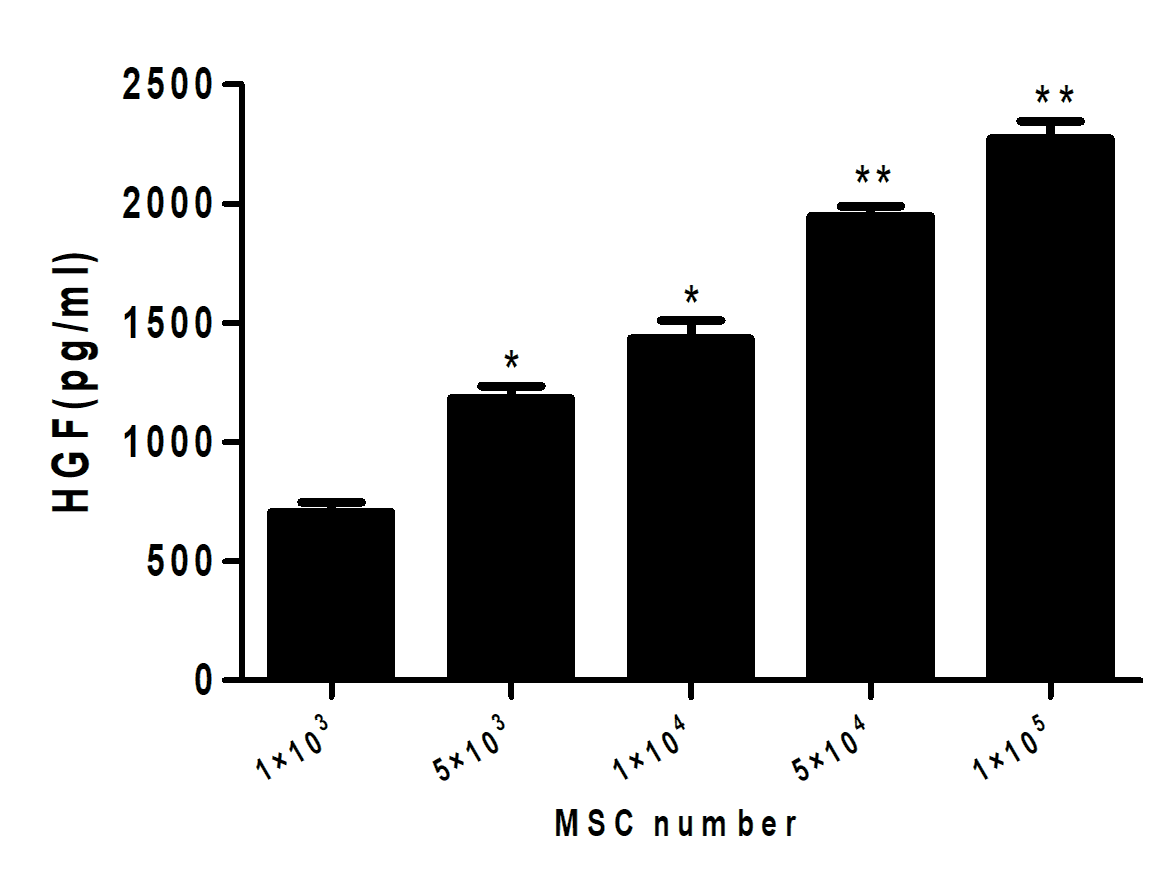

Supplement: Supplementary file 2 — Additional file 2. Effects of different numbers of MSCs on secreted HGF levels measured by ELISA. MSCs, mesenchymal stem cells; rmHGF, recombinant mouse hepatocyte growth factor; ELISA, enzyme-linked immunosorbent assay. [file 13287_2020_1612_MOESM2_ESM.tif]
